# Genotypic Characterization of Uropathogenic *Escherichia coli* from Companion Animals: Predominance of ST372 in Dogs and Human-Related ST73 in Cats

**DOI:** 10.3390/antibiotics13010038

**Published:** 2023-12-30

**Authors:** Sophie Aurich, Silver Anthony Wolf, Ellen Prenger-Berninghoff, Lakshmipriya Thrukonda, Torsten Semmler, Christa Ewers

**Affiliations:** 1 Institute of Hygiene and Infectious Diseases of Animals, Faculty of Veterinary Medicine, Justus Liebig University Giessen, 35392 Giessen, Germany; ellen.prenger-berninghoff@vetmed.uni-giessen.de (E.P.-B.); christa.ewers@vetmed.uni-giessen.de (C.E.); 2 Genome Competence Centre, Robert Koch Institute, 13353 Berlin, Germanythrukondal@rki.de (L.T.);

**Keywords:** ExPEC, UPEC, dog, cat, antimicrobial resistance genes, phylogroup B2, ST73, ST372

## Abstract

Extraintestinal pathogenic *Escherichia coli* (ExPEC) account for over 80% and 60% of bacterial urinary tract infections (UTIs) in humans and animals, respectively. As shared uropathogenic *E. coli* (UPEC) strains have been previously reported among humans and pets, our study aimed to characterize *E. coli* lineages among UTI isolates from dogs and cats and to assess their overlaps with human UPEC lineages. We analysed 315 non-duplicate *E. coli* isolates from the UT of dogs (198) and cats (117) collected in central Germany in 2019 and 2020 utilizing whole genome sequencing and in silico methods. Phylogroup B2 (77.8%), dog-associated sequence type (ST) 372 (18.1%), and human-associated ST73 (16.6%), were predominant. Other STs included ST12 (8.6%), ST141 (5.1%), ST127 (4.8%), and ST131 (3.5%). Among these, 58.4% were assigned to the ExPEC group and 51.1% to the UPEC group based on their virulence associated gene (VAG) profile (ExPEC, presence of ≥VAGs: *papAH* and/or *papC*, *sfa*/*focG*, *afaD*/*draBC*, *kpsMT*II, and *iutA;* UPEC*,* additionally *cnf1* or *hlyD*). Extended-spectrum cephalosporin (ESC) resistance mediated by extended-spectrum β-lactamases (ESBL) and AmpC-β-lactamase was identified in 1.9% of the isolates, along with one carbapenemase-producing isolate and one isolate carrying a *mcr* gene. Low occurrence of ESC-resistant or multidrug-resistant (MDR) isolates (2.9%) in the two most frequently detected STs implies that *E. coli* isolated from UTIs of companion animals are to a lesser extent associated with resistance, but possess virulence-associated genes enabling efficient UT colonization and carriage. Detection of human-related pandemic lineages suggests interspecies transmission and underscores the importance of monitoring companion animals.

## 1. Introduction

Extraintestinal pathogenic *Escherichia coli* (ExPEC) are recognized as the leading cause of community-acquired and hospital-acquired urinary tract infections (UTIs) in both humans and companion animals [1,2,3,4]. Other significant pathogens commonly found include *Staphylococcus pseudintermedius*, *Enterococcus faecalis*, and various *Enterobacterales*, such as *Proteus* sp., *Klebsiella* sp., and *Enterobacter* sp. Additionally, *Streptococcus canis* and *Pseudomonas aeruginosa* have been described as being responsible for UTI infections in companion animals [2]. UTI caused by *E. coli* is one of the most common indications for which antibiotic treatment is initiated [5]. These treatments, however, have faced significant challenges due to the increase of multidrug-resistant (MDR) bacteria, including extended-spectrum β-lactamase (ESBL)-producing bacteria and isolates carrying plasmid-mediated cephalosporinases [6,7,8]. ESBL-producing *E. coli* show resistance to extended-spectrum cephalosporins (ESCs) but are still susceptible to β-lactam/β-lactamase inhibitor combinations [9]. In contrast, plasmid-mediated cephalosporinases remain unaffected by β-lactam/β-lactamase inhibitor combinations [10]. Moreover, fluoroquinolone resistance further narrows the choice of antimicrobial treatment of UTIs caused by *E. coli* or other bacterial pathogens. Fluoroquinolone resistance mechanisms include mutations in the quinolone resistance-determining region of bacterial type-II topoisomerase genes *gyrA* and *parC* or the presence of plasmid-encoded genes *qnr* and *aac(6′)-Ib-cr* [11]. As a result, the efficacy of commonly utilized antimicrobials like enro- and pradofloxacin, cefovecin, and amoxicillin/clavulanic acid is frequently impaired [12,13].

The majority of UTIs in humans and animals are caused by ExPEC, particularly the uropathogenic *E. coli* pathotype (UPEC). Both can cause extraintestinal disease in otherwise healthy individuals, including meningitis, intra-abdominal infection, pneumonia, and UTI, while being able to persist in the gut without causing inflammation [1,14,15,16,17]. Due to the presence of a distinctive set of virulence associated genes (VAGs), these pathotypes possess an enhanced ability to overcome host defences. VAGs coding for adhesins, toxins, and protectins allow ExPEC and UPEC to successfully colonize the urinary bladder, damage epithelial cells, and survive in the urinary tract. Additionally, they have evolved metabolic adaptations, such as enhanced iron acquisition systems, in order to survive across nutrient-scarce environments [18,19,20,21]. The distinction of ExPEC and UPEC from other *E. coli* pathotypes is based on the presence of these VAGs, which follow different classification schemes [22,23,24]. In 2000, Cermont et al. identified four phylogenetic groups within *E. coli* strains using PCR based on the presence of specific genes [25]. Currently, eight distinct phylogroups (A, B1, B2, C, D, E, F, and G) and various *E. coli* clades are recognized [26]. ExPEC and UPEC have mainly been associated with phylogroup B2, and to a lesser extent with phylogroups D and F [27,28].

Studies on the population structure of UPEC in dogs predominantly revealed multilocus sequence type ST372, as well as ST12, ST73, ST127, ST131, and ST141 [29,30,31]. Notably, all of these STs, with the exception of ST372, have also been associated with UTI in humans [32,33]. Few studies are available on the population structure of UPEC in cats, which mainly identified ST73 and ST83 [6,8,34,35]. Certain clonal linages appear to be more successful in causing extraintestinal infections and disseminating among certain host populations. Some clonal lineages have also been associated with the carriage of ESBLs and/or with fluoroquinolone resistance, such as O25b:H4-B2-ST131 or O6:H1-B2-ST73 [36]. With pets increasingly becoming integrated into human households, the risk of cross-transferring antimicrobial-resistant (AMR) bacteria and/or AMR genes between pets and their owners has emerged as a public health concern [37,38,39]. Monitoring the spread of these clones might essentially contribute to combating the global spread of ExPEC.

Therefore, we performed an in-depth analysis of UTI-causing *E. coli* isolated from routine diagnostic specimens of dogs and cats with symptoms of UTIs. We provide data on the pathotypes, phylogroups, serotypes, and clonal structure, and insights into the distribution of the VAG and AMR genes.

## 2. Results

### 2.1. Bacterial Isolates

We obtained 315 unique *E. coli* isolates from 195 dogs (63.3%) and 113 cats (36.7%) from 33 veterinary clinics during routine microbiological diagnostics. All samples were submitted with a preliminary report of suspected bacterial urinary tract infection. The sample pool consisted of urine samples (98.1% [cystocentesis, 17.5% midstream voided samples, 5.2% catheter specimens, 50.5% unspecified sampling procedure]) and, to a lesser extent (1.9%), of bladder tissue, uricult tests, uroliths, and kidney swabs. Notably, 48.9% of the isolates were provided by only three distinct veterinary clinics. A haemolytic phenotype was identified in 194 (61.6%) isolates. In 28.9% of the cases, the isolates were obtained from mixed infections, i.e., together with other specific uropathogens or nonspecific bacteria in low bacterial counts. The remaining 71.1% of isolates were obtained from monoinfections in pure culture.

### 2.2. Clonal Typing of E. coli Isolates

Phylogenetic group B2, which is associated with extraintestinal and uropathogenic *E. coli* infections in humans and animals, was the most frequently identified phylogroup (77.8%) (Table 1). Phylogroups D and F, also associated with extraintestinal infections, accounted for 2.2% and 0.6%, respectively.

Based on multilocus sequence typing (MLST), 315 isolates were assigned to 82 distinct sequence types (STs), with ST372 (17.5%), ST73 (15.9%), ST12 (8.6%), ST141 (4.8%), ST127 (4.8%), and ST131 (3.5%) being the most prevalent. Ten allele profiles (3.2%) could not be assigned to existing STs and were submitted to Enterobase (https://enterobase.warwick.ac.uk/; accessed on 19 September 2023) for further characterization (Supplementary Table S1). The distribution of ST372 and ST73 differed significantly between dogs and cats: ST372 was more prevalent in dogs (26.8%) compared to cats (1.7%) (*p* < 0.001), while ST73 was more common in cats (27.4%) than in dogs (9.1%) (*p* = 0.0002). ST73 (*p* < 0.001), ST372 (*p* = 0.018), ST12 (*p* = 0.001), and ST127 (*p* = 0.006) were positively associated with the haemolytic phenotype. A total of 32 different O-serogroups were identified among the *E. coli* isolates. Among the type I fimbrial adhesion gene alleles, *fimH*9 was the most frequently detected *fimH* type (20.3%) predominantly linked with ST372 (85.4%) (*p* < 0.0001). In cases of the second most common ST73, both *fimH*9 (28.0%) and *fimH*102 (18.0%) were the predominant *fimH* types.

**Figure 1:**
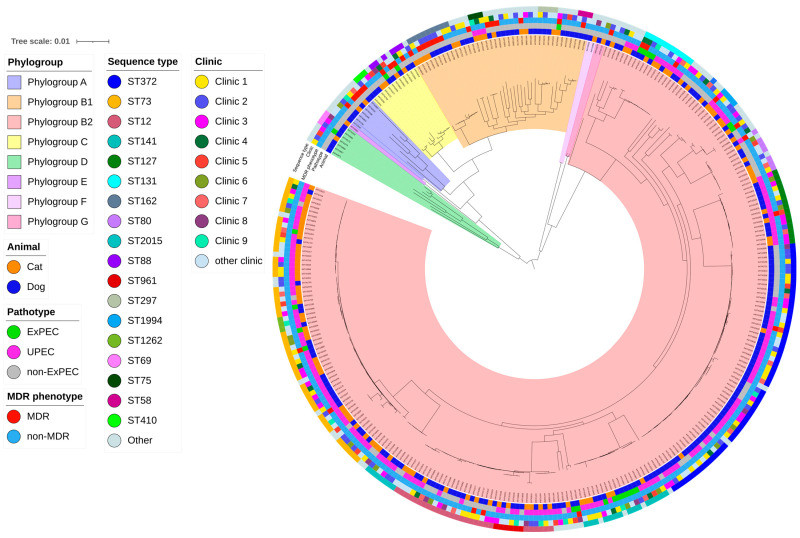
Core gene-based maximum-likelihood phylogenetic tree of 315 *E. coli* isolates from the urinary tract of cats and dogs. The inner to outer coloured rings display additional metadata defined by the host species, pathotype, phylogroup, sequence type, putative MDR phenotype, and site of isolation.

Based on the presence of the VAGs *papAH* and/or *papC*, *sfa*/*focG*, *afaD*/*draBC*, *kpsMT*II, and *iutA*, 58.4% of the isolates were assigned to the ExPEC group. Among all isolates, 51.1% were classified as UPEC as they additionally possessed *cnf1* or *hlyD*. Nearly all (98.1%) UPEC isolates were assigned to phylogroup B2 (Figure 1). In cases of mixed infections, *E. coli* isolates were less frequently classified as ExPEC (*p* = 0.04). The results of the sero(geno)typing and typing of *fimH* are displayed in Table 1.

### 2.3. Distribution of Virulence-Associated Genes

Virulence genotyping revealed a high number of VAGs required for colonizing the urinary tract, overcoming host immunity responses, and persisting in the urinary tract. The most abundant VAGs were genes encoding type 1 fimbriae (*fimACDH*, 98.7%), flagella (*flg*BCDEFGHIJ**, 99.7%), and curli (*csgABC/DEFG*, 100%). Those factors play a crucial role in *E. coli* biofilm formation by mediating attachment to epithelial cells in the urinary tract [20]. In addition, genes related to the pathogenicity island marker *MalX*, which possesses glucose and maltose transporting activity, and the outer membrane protein OmpA, which promotes persistence of UPEC in the bladder, were present throughout the isolate collection (100%, each) [40]. Genes coding for the siderophore systems enterobactin (*ent*/*fep*/*fes*; 99.7%), yersiniabactin (*irp/fyuA;* 85.4%), and salmochelin (*iroBCDE/N*; 86.8%) were highly abundant, while aerobactin genes (*iuc/iutA;* 15.2%) occurred less frequently. Certain VAGs, such as the P fimbriae adhesion gene *papG* allele III, hemolysin gene *hly*, and cytotoxic necrotizing factor gene *cnf1*, have previously been associated with canine and feline UTIs and were accordingly detected in 45.1%, 46.3%, and 57.8% of our isolates, respectively [41]. A detailed distribution of the ExPEC-associated VAGs is provided in Table 2. In addition, a comprehensive listing of the VAG distribution among the bacterial isolates is shown in Table S2.

**Figure 2:**
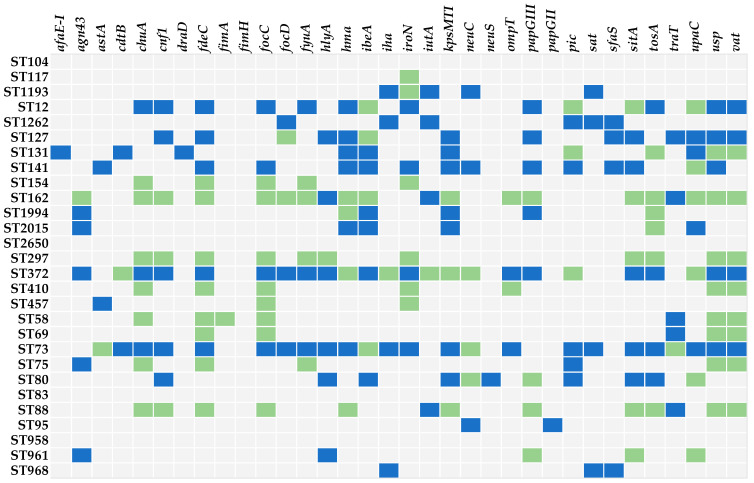
Correlations among 34 VAGs identified in UPEC isolates and 26 dominant STs: blue coloured cells indicate a significant positive association between VAG and ST (*p* ≤ 0.05), light grey coloured cells indicate no significant association (*p* > 0.05), and green coloured cells indicate a significant negative association (*p* ≤ 0.05) as determined by Chi-square tests.

Positive correlations between the presence of UPEC-specific VAGs and STs were most frequently observed for the prevailing B2-STs, namely ST73, ST372, ST141, ST127, and ST12 (Figure 2). Conversely, B1-ST162 and B1-ST297, along with C-ST88, revealed negative associations with UPEC-specific VAGs. In contrast to other phylogenetic groups, B2 isolates more frequently revealed virulence factors that contribute to colonization and pathology of the urinary tract and to bacterial persistence, suggesting that B2 isolates are generally more successful in causing UTIs.

### 2.4. Distribution of AMR Genes

Among the 315 *E. coli* isolates, only 89 isolates, obtained from 51 dogs and 38 cats, possessed AMR genes. In total, 373 different AMR genes were identified (Table 3). Of these, *bla*_TEM-1B_ represented the most common one (49.4%), followed by *sul2* (41.6%) and *tet(A)* (32.6%). A detailed distribution of the resistance genes is shown in Table S3. There was no correlation between the occurrence of AMR genes and animal species. However, a lower number of AMR genes was present in the most prevalent phylogroup B2 compared with other phylogenetic groups. This was particularly notable concerning folate pathway inhibitors, including *sul1*, *sul2*, *sul3*, and different variants of *dfrA* (Table 3).

Non-susceptibility to fluoroquinolones (FQ) was demonstrated in 26 (8.2%) of the isolates. Susceptibility testing was conducted during a previous study (see [2] for the methodology). Genotyping revealed plasmid-located genes *qnrS1*, *aac(6’)-Ib-c*, *qnrS2*, *qnrB19*, and *qnrB4* (Table 2) in five of these isolates. Furthermore, all FQ non-susceptible isolates showed mutations in the quinolone resistance-determining region, namely S83L in *gyrA* (7.7%), and S83L and D87N in *gyrA* (92.3%). In 96.2% of the FQ resistant isolates, an additional mutation in *parC* (S80I) was observed (Table S3).

Resistance to ESC was also demonstrated in a previous study: 2.9% (n = 9) of the 315 isolates were non-susceptible to cefovecin, a third-generation cephalosporin [2]. Genotypic analysis revealed that four of these isolates carried an ESBL (3 × CTX-M-15 and 1 × CTX-M-27), and two carried an AmpC-β-lactamase (1 × CMY-2 and 1 × DHA-1). Additionally, one canine ST58 ESC-resistant isolate showed chromosomal mutations that resulted in amino acid substitutions of the *ampC* promotor region (C → T at position −42). This is the most frequently reported promotor mutation; it is associated with a 20-fold increase in enzyme production [42]. The same isolate also harboured a chromosomally encoded ES class C beta-lactamase *bla*_EC_ gene_,_ which was closely related to *bla*_EC11_ (99.2%) and its encoded protein (98.7%) [43]. One isolate carried ES class C beta-lactamase *bla*_EC-6_ [43]_._ An overview of the ESC-resistant isolates is provided in Table 4.

Multi-drug resistant (MDR) isolates, i.e., isolates non-susceptible to at least one antibiotic agent in three or more categories, were identified in 11.7% of 315 UTI isolates in our previous study [2]. MDR isolates were obtained in nearly equal proportions from dogs (n = 25; 12.6%) and cats (n = 12; 10.3%) (Figure 1). Only two STs significantly more often displayed MDR isolates: ST162 (*p* < 0.001; 66.7%; dog, n = 4; cat, n = 2) and ST88 (*p* = 0.002; 50.0%; dog, n = 2; cat, n = 1). Of the dominant ST372 and ST73 isolates in this study, only one and two, respectively, showed an MDR phenotype. The single ST14 isolate exhibited MDR, while ST1193, which is a single-locus variant of ST14, was only FQ- and ampicillin-resistant. Of 11 ST131 isolates, three isolates (27.3%) were MDR. A detailed comparison of the AMR genes and the corresponding phenotypic resistance is provided in Supplementary Table S3. A combination of serogroup O25b:H4 and ST131 was identified in 3.5% of our isolates. Generally, ST131 is divided into three clades with different *fimH* allelic variants, i.e., A/*H*41, B/*H*22, and C/*H*30, with clade C being by far the most represented among human and animal ExPEC isolates. Within clade C, two subclades are defined: C1/*H*30R1, associated with fluoroquinolone resistance conferred by mutations in the chromosomal genes *gyrA* and *parC*, and C2/*H*30Rx, associated additionally with ESC resistance [44,45]. Only two isolates from our study displayed fluoroquinolone resistance, and they possessed the *fimH* allelic variant H30 and were therefore assigned to clade C. As none of them showed additional ESC resistance, they were grouped into subclade C1/H30R1. Four of the ST131 isolates possessed *fimH*22 and another four isolates harboured *fimH*298, which differs from *fimH*22 by only a single nucleotide [46]. Thus, these eight isolates can be assigned to clade B.

## 3. Discussion

In order to investigate the population structure of *E. coli* isolates causing clinical symptoms of UTIs in dogs and cats, we conducted a comprehensive genomic analysis of 315 isolates (198 of canine and 117 of feline origin). The isolates were collected from submissions to our diagnostic department, due to diagnosis of suspected bacterial UTI. As to be expected, the majority of the isolates (77.8%) was assigned to phylogroup B2. This phylogroup has been frequently associated with ExPEC, and especially with isolates from the urinary tract of humans and animals [31,33,47,48,49]. We demonstrated a positive correlation between phylogroup B2 and the VAG-based categorisation of isolates as ExPEC. In detail, 69.1% of *E. coli* group B2 isolates from dogs and 77.4% from cats were classified as ExPEC (*p* < 0.001). Additionally, regarding the B2 isolates, 59.2% from dogs and 72.0% from cats were further identified as UPEC (*p* < 0.001), which corroborates previous data obtained by comparable methods [31,47].

Based on the presence of specific VAGs, only 51.1% and 57.8% of the isolates were categorized as UPEC and ExPEC, respectively [22,23]. The relatively low number might be considered somewhat surprising given that all isolates were obtained from the urinary tract of animals exhibiting symptoms of UTIs. It has to be considered though, that in cases of underlying comorbidities or the use of medical devices such as urinary catheters, opportunistic *E. coli* isolates, lacking a specific set of VAGs, might likewise be capable of causing UTIs. Notably, when *E. coli* was classified as ExPEC, it was often the sole causative agent obtained from the site of infection. In contrast, isolates were less frequently classified as ExPEC in cases of mixed infections.

The distribution of UPEC-associated *papG* alleles, which encode for the adhesin of P fimbriae, showed a clear predominance of *papG*III (45.1%), which is commonly associated with acute cystitis in both humans and dogs. In contrast, *papG*II, which has been linked to human pyelonephritis, was less prevalent [41]. Interestingly, *papG*III showed a positive association with the presence of *cnf1* and *hlyA* genes (*p* < 0.001), indicating a potential correlation between these VAGs. On the other hand, *papG*II did not exhibit any association with *hlyA* and was negatively associated with *cnf1* (*p* = 0.008). Furthermore, we demonstrated that B2 isolates harbour fewer AMR genes but are more prone to carry UPEC associated VAGs. These data substantiate the hypothesis previously established by various authors: that the acquisition of AMR genes may induce the loss of virulence factors [21,50]. For example, mutations in the chromosomal coded *gyrA* gene of UPEC have been associated with reduced bacterial virulence. One explanation is that during the development of FQ resistance, increased deletion and transposition of DNA regions, such as pathogenicity islands (PAIs), is achieved [51]. PAIs consist typically of VAGs encoded within chromosomal regions and are capable of being exchanged via horizontal gene transfer [52]. Among our isolates, we detected *malX* and *usp,* located on PAI I_CFT073_ and PAI*_usp_*, in 100% and 75.9% of the isolates, respectively [53,54]. *Usp* has been associated with UTI isolates and is suggested as a potential VAG for use as a molecular epidemiological marker specific for UPEC. *MalX*, although located on the same PAI as genes encoding α-haemolysin, P-fimbriae, and aerobactin, which are established contributors to virulence, currently lacks a known role in virulence [54,55].

Another explanation for the hypothesis that the acquisition of AMR genes induces loss of virulence is the concept of plasmid competition, wherein plasmids carrying resistance or virulence factors compete with each other [56]. However, it is important to note that the acquisition of AMR genes does not necessarily equate to the loss of VAGs. This phenomenon can vary depending on the mechanism of resistance and the antibiotic studied [50].

With 2.5% ESBL- or AmpC-producing isolates, we observed a lower prevalence than found in most previous studies [57,58,59,60]. However, the studies cited investigated not only isolates from UTI but also from other sites of infection. Only four isolates harboured a CTX-M-type enzyme, namely CTX-M-15 (3×) and CTX-M-27 (1×). CTX-M-15 is the predominant ESBL type in *E. coli* isolated from companion animals in Europe and the American continent [38,60]. The AmpC-β-lactamase CMY-2, which is considered the most common AmpC type currently encountered in humans and animals, was only isolated once [10,38,60]. We also found one *bla*_DHA-1_ and two *bla*_EC_ genes. It remains unclear whether *bla*_EC_ confers ESC resistance since additional mutations in the *ampC* promoter (IHIT42298, −42C > T, −18G > A, −1C > T; IHIT44091, −28G > A) were observed in both isolates. Whilst *bla*_DHA_ has been found in *E. coli* obtained from urine samples of companion animals, there have been no studies describing the presence of *bla*_EC_ in uropathogenic *E. coli* [60].

MDR isolates (dog, n = 25; cat, n = 11) were found more often in non-B2 phylogroup isolates than in B2 isolates (dog, 40.0%; cat, 27.3%; *p* < 0.001), namely phylogroup A (dog, 16.0%; cat, 9.1%), B1 (dog, 28.0%; cat, 54.6%), and C (dog, 16.0%; cat, 9.1%). These findings may favour more effective antimicrobial therapy of UTIs and/or reduced treatment failures, considering that the majority of UTI cases are caused by B2 isolates.

The occurrence of the carbapenem (CP) resistance gene *bla*_OXA-48_ in *E. coli* from companion animals, particularly in cats, has been rarely documented. In Germany, few cases of OXA-48-producing *E. coli* in dogs have been reported and, to date, only one OXA-48 positive isolate has been published from a cat [61,62,63]. However, our group has identified additional OXA-48-positive clinical *E. coli* isolates from cats along with another study (unpublished data). Since carbapenems are not approved for veterinary use, it has been hypothesized that the occurrence of CP resistant *Enterobacterales* in animals may be attributed to a zoonotic transmission of bacteria and/or genetic determinants from humans to animals [61,64]. The mobile colistin resistance gene *mcr*-4.6 was identified in the canine ST73 isolate IHIT43641, located on a ColE10 plasmid. To date, only a limited number of MCR-carrying bacteria have been detected in companion animals in Europe. Colistin (polymyxin E), a cationic antimicrobial peptide that entered clinical use in 1959, was abandoned in the 1980s because of its neurotoxic and nephrotoxic effects [65]. However, with the emergence of MDR gram-negative bacteria, colistin was reintroduced in the human domain. In veterinary medicine, it has been widely used, particularly for the treatment of neonatal and post-weaning diarrhoea in pigs and gastrointestinal infections of calves [66,67,68]. Several studies suggest the origin of these resistance genes in the easily transferable mobile elements originating from isolates in the environment and from farm animals, since the treatment of companion animals with colistin is not common in Europe (reviewed in [69]).

The most frequently identified STs, namely ST372 and ST73, displayed an animal species-specific distribution pattern: ST372 was almost exclusively isolated from dogs, whereas a particularly high prevalence of ST73 was observed among isolates from cats. This host-specific distribution has also been documented by other researchers, suggesting that there might be an adaptation of certain lineages to UTI infections in either dogs or cats [29,30,31,70,71]. Similar to Zogg at al., 2018, we found no correlation between the two most common STs and MDR isolates or strains carrying ESBL or AmpC-β-lactamase [8]. The association of ST372 with dogs has recently been mentioned in the context of commercially available dog food. Elankumuran et al., 2023, identified the genes of the propanediol utilization (*pdu*) operon as accessory genes in ST372 *E. coli* isolates from dogs. This operon was originally described for its role in microcompartment-mediated metabolism of glycerol and 1,2-propanediol in *Salmonella enterica* serovar Typhimurium and has been associated with gastrointestinal colonization and pathogenicity. The authors raised the question of whether the high prevalence of ST372 isolates in dogs could be attributed to the presence of glycerol and 1,2-propanediol, which are common additives in semi-moist commercially available dog food [71]. However, it remains unclear why cats, which are also fed commercially available food, predominantly exhibit a high number of ST73 isolates and are rarely infected with ST372 isolates.

Despite the heterogeneity of STs observed in the majority of isolates, a notable finding is the presence of several STs typically associated with humans, indicating a significant overlap between species. The success of certain clonal lineages isolated from humans and animals alike, such as the ESBL-producing *E. coli* strain O25b:H4-ST131 or the emerging high-risk clone ST410, have been well documented [72,73,74]. O25b:H4-ST131 was identified in 3.5% of our isolates; two of them were assigned to subclade C1/H30R1 and eight isolates to clade B. Three of these isolates showed an MDR phenotype. Interestingly, none of the them harboured an ESBL, although the emergence of ST131 possessing a CTX-M gene has been reported frequently among humans and companion animals lately [44,46,72,75]. Two isolates were assigned to ST1193, previously determined as the FQ-resistant B2 *E. coli* group, which is referred to as a pandemic ST by Kidsley et al., 2020 [75]. Tchesnokova et al., 2019, identified this ST in one quarter of human clinical urine isolates in the USA, and also in Germany, ST1193 was increasingly detected over the last years in humans [76,77]. In our study, ST1193 was relatively rare, accounting for only 0.6% of isolates. Fluoroquinolone-resistant *E. coli* assigned to ST410, an “international high-risk clone” for AMR, were found in three dogs [74,78]. One ST410 additionally carried the ESBL CTX-M-15. The overall low occurrence of pandemic AMR clones, such as ST410, ST1193, and ST131 in our study population, and in companion animals in general, may suggest that the primary source of these STs are humans. Companion animals may be more likely to serve as spillover hosts than the original source of these STs known for their increased resistance [75].

Limitations of this study include the missing data on antibiotic treatment prior to sampling. Since a large proportion of the samples originated from only three veterinary clinics (specialist referral clinics), it cannot be ruled out that our data are biased towards complicated cases of cystitis. A separate study with a detailed preliminary report concerning symptoms and treatments would be beneficial to get an even deeper insight into the relevance of distinct pathovars, STs, and VAGs of *E. coli* isolates associated with complicated and uncomplicated urinary tract infections.

## 4. Conclusions

The majority of *E. coli* isolates obtained from dogs and cats with symptoms of urinary tract infections were assigned to phylogroup B2. Multidrug-resistant and ESBL/AmpC-producing isolates were less often found in this phylogroup and in general in our study population compared with other studies. Only a small proportion of isolates were assigned to so-called “high-risk clones” for AMR. Nevertheless, pandemic ExPEC lineages ST69, ST73, ST95, ST127, and ST131 were also present among our isolates, indicating the wide distribution of human-related ExPEC/UPEC strains in cats and dogs. Further surveillance of *E. coli* clonal lineages implicated in UTIs in companion animals is warranted, as the transmission of ESC-resistant *E. coli* between dogs and humans, particularly within households, has been well-documented [79,80,81,82,83]. Understanding transmission dynamics is crucial to assess the potential impact on public and animal health. Nonetheless, the AMR data gained in this study might be considered as a positive indication in the context of the *One Health* approach and, in a clinical context, they suggest that a high proportion of UTIs caused by *E. coli* in companion animals can still be effectively treated with antibiotics.

## 5. Materials and Methods

### 5.1. Sampling and Identification of Bacterial Isolates

*Escherichia coli* isolates were collected from the urinary tract of cats and dogs between November 2019 and November 2020. The isolates were obtained from routine diagnostic submissions to the veterinary diagnostic laboratory of the Institute of Hygiene and Infectious Diseases of Animals, Department of Veterinary Medicine, Justus Liebig University Giessen, Germany. Samples consisted of urine and of bladder swabs/bladder tissue, prostate swabs, uricult tests, uroliths, and kidney swabs. Samples were streaked out on standard nutrient agar (Oxoid, Wesel, Germany) supplemented with 5% defibrinated sheep blood and on water-blue metachrome-yellow lactose agar according to Gassner (sifin diagnostics gmbh, Berlin, Germany) and incubated under aerobic conditions at 37 °C. In cases where multiple samples were submitted for one animal during the study period, all samples were subjected to further phenotypic and genotypic analysis. However, only the first sample was considered for the final analysis, unless changes in the AMR and VAG profile based on whole genome data indicated a non-duplicate strain or the acquisition of additional resistance genes. Generally, a single colony was collected and analysed as representative for the bacterial population causing the infection. The haemolytic phenotype of *E. coli* was investigated on sheep blood agar. In cases where both haemolytic and non-haemolytic phenotypes were cultured from the same sample, a representative colony of both was collected and further analysed.

Species identification was performed using matrix-assisted laser desorption time-of-flight mass spectrometry (MALDI-TOF MS, Microflex, Bruker Daltonics, Bremen, Germany) [84]. Therefore, a single colony was smeared onto a polished steel MALDI target plate (Bruker Daltonics, Bremen, Germany) and allowed to dry at room temperature. The sample was overlaid with 1 µL matrix (Bruker Daltonics). Mass spectra were acquired using a mass range of 2–20 kDa using Biotyper version 3.3.1.0. (Bruker Daltonics). Species identification was considered valid at score values > 2000 according to the manufacturer’s instructions (database v9.0.0.0). Metadata including host species, age, and sex were extracted from the laboratory submission form or from practice management software.

### 5.2. Isolate Storage, DNA Preparation, and Whole Genome Sequencing

All isolates were stored in Brain Heart Infusion Broth (Oxoid, Wesel, Germany) with 30% glycerol at −70 °C. Genomic DNA was extracted using the Master Pure™ DNA Purification Kit (Biozym Scientific GmbH, Hessisch Oldendorf, Germany). Bacterial genomes were sequenced using an Illumina MiSeq sequencer (MiSeq Reagent Kit V.3; Illumina Inc., San Diego, CA, USA) with multiplexing of 30 samples per flow cell using 2 × 150 bp paired-end reads to obtain an average of 90-fold coverage. Quality control and adapter trimming were performed by an in-house pipeline. De novo assemblies were generated by the SPAdes Genome Assembler (v3.15.5) [85]. The Bakta pipeline (v1.8.2) was employed for genomic annotation of the bacterial genomes [86].

### 5.3. In Silico Methods

Pathotype assignment was carried out based on published criteria: *E. coli* isolates harbouring two or more of the VAGs *papAH* and/or *papC*, *sfaS*/*focG*, *afaD*/*draBC*, *kpsMT*II, and *iutA* were classified as ExPEC [22]. If toxin and hemolysin genes *cnf1* and *hlyD* were also present, the isolate was assigned to the UPEC pathotype [23]. Virulence genotyping was performed using VirulenceFinder 2.0 hosted by the Center for Genomic Epidemiology (https://cge.food.dtu.dk/services/VirulenceFinder/, accessed on 7 June 2023). Additionally, a custom in-house database was created based on 551 VAGs described for various *E. coli* pathovars in public databases such as CARD, Institut Pasteur and NCBI (https://www.ncbi.nlm.nih.gov/nucleotide/, https://bigsdb.pasteur.fr/ecoli/, https://card.mcmaster.ca/home, accessed on 22 June 2023). This custom collection was utilized with the ABRicate software (v1.0.1, https://github.com/tseemann/abricate, accessed on 29 June 2023) using a threshold of 80%, or 99% in cases of screening for specific alleles, to profile the corresponding assemblies. MLST 2.0 (https://cge.food.dtu.dk/services/MLST/, accessed on 11 July 2023) was used to determine sequence types (STs) according to the Achtman scheme, employing seven housekeeping genes (*adk*, *fumC*, *gyrB*, *icd*, *mdh*, *purA*, and *recA*). Sero(geno)typing (O:H) was conducted using SerotypeFinder 2.0 (https://cge.food.dtu.dk/services/SerotypeFinder/, accessed on 14 June 2023). Typing of *fimH* was achieved by using FimTyper 1.1 (https://cge.food.dtu.dk/services/FimTyper/, accessed on 15 June 2023). AMR genes and chromosomal point mutations were determined using ResFinder 4.1 (https://cge.food.dtu.dk/services/ResFinder/, accessed on 21 June 2023) and BacWGSTdb (http://bacdb.cn/BacWGSTdb/analysis_single.php, accessed on 11 July 2023). All *bla*_TEM_ and *bla*_SHV_ genes were further determined by the lactamase engineering database (http://www.laced.uni-stuttgart.de/, accessed on 25 January 2023).

The isolates were also classified into one of the eight *E. coli* phylogenetic groups (A, B1, B2, C, D, E, F, and G) or a cryptic clade using the refined ClermonTyping method, based on the in vitro PCR assay developed by Clermont et al., 2000, targeting *chuA*, *yjaA*, TspE4.C2, *arpA,* and *trpA* (http://clermontyping.iame-research.center/, accessed on 12 August 2023).

The population structure of the sample collection was further investigated through phylogenetic reconstruction of a maximum likelihood tree. Here, a gene-by-gene approach was first utilized to establish a shared set of core genes. This was generated using the annotated bacterial assemblies in combination with the roary software (v3.13.0) [87]. A total of 2820 conserved genes were identified using this approach. They were present in at least 99% of the strains (protein sequence similarity min. 95%, sequence coverage min. 90%). This was followed by gene-wise alignments via the Mafft software (v7.520) [88] and subsequent concatenation of the alleles per sample. The resulting alignment was then used to infer a phylogeny through RAxML-NG (v.1.2.0) [89] with a General Time Reversible model and gamma correction for among site rate variation. Finally, iTOL (v6.8.1) [90] was utilized to visualize the population structure in the context of the available metadata.

### 5.4. Statistical Analysis

Statistical analysis was carried out using the SAS 9.4 statistical software package [91]. Descriptive statistics were performed for all data. Categorical parameters were compared between groups using a chi-square test. In instances where the chi-square test was not applicable due to low frequencies (more than 20% of the cells had expected frequencies below [92]) a Fisher exact test was employed. The significance level for all statistical analyses was established at *p* < 0.05.

## Figures and Tables

**Table 1:** Results of phenotype, clonal typing, sero(geno)typing, and *fimH-*typing of 198 canine and 117 feline *E. coli* isolates.

	Phylogenetic Group	Haemolytic Phenotype	Multilocus Sequence Type	Pathotype	Sero(geno)type	FimH Type
	(percentages of positive isolates are given in brackets)					
Dog	A (3.0) B1 (12.1) B2 (76.8) C (4.0) D (3.0) E (0.0) F (0.5) G (0.5)	non-haemolytic (43.4) haemolytic (56.6)	ST372 (26.8) ST73 (9.1) ST12 (8.1) ST127 (5.1) ST141 (4.6) ST131 (3.0) ST162 (2.5) ST80 (1.52) ST2015 (2.53) Other (36.9)	ExPEC (8.6 *) UPEC (46.0) Other (45.5)	Ont (24.8) O4 (17.2) O2 (13.1) O6 (10.6) O25 (3.5) O8 (4.6) O83 (5.1) O15 (5.1) O25b (2.5) O9 (2.5) Other (11.1)	*fimH*9 (25.9) *fimH*5 (5.1) *fimH*32 (4.6) *fimH*2 (3.6) *fimH*102 (3.1) *fimH*13 (0.5) *fimH*27 (2.5) *fimH*14 (2.5) *fimH*197 (2.5) *fimH*1 (2.0) *fimH*39 (2.0) *fimH*31 (2.6) Other (44.2)
Cat	A (1.7) B1 (12.0) B2 (79.5) C (3.4) D (0.8) E (0.8) F (0.8) G (0.8)	non-haemolytic (29.9) haemolytic (70.1)	ST372 (1.7) ST73 (27.4) ST12 (9.4) ST127 (4.3) ST141 (5.1) ST131 (4.3) ST162 (3.4) ST80 (3.4) ST2015 (1.7) Other (39.3)	ExPEC (5.1 *) UPEC (59.8) Other (35.0)	Ont (26.5) O4 (11.1) O2 (17.1) O6 (8.6) O25 (12.0) O8 (5.1) O83 (1.7) O15 (0.0) O25b (3.4) O9 (3.4) Other (11.1)	*fimH*9 (11.1) *fimH*5 (5.1) *fimH*32 (5.1) *fimH*2 (6.0) *fimH*102 (3.4) *fimH*13 (6.0) *fimH*27 (2.6) *fimH*14 (1.7) *fim*197 (1.7) *fimH*1 (1.7) *fimH*39 (1.7) *fimH*31 (2.6) Other (51.3)

* Indicates the number of isolates that could be assigned to the pathotype ExPEC but were not confirmed as UPEC.

**Table 2:** Distribution of ExPEC-associated genes among 315 *E. coli* isolates from 198 dogs and 117 cats.

Category	Gene	Dog N (%)	Cat N (%)	Total N (%)	*p*-Value ^1^
Adhesins					
Fimbrial	*papG*I	1 (0.5)	3 (2.6)	4 (1.3)	0.146
	*papG*II	5 (2.5)	2 (1.7)	7 (2.2)	0.483
	*papG*III	92 (46.5)	50 (42.7)	142 (45.1)	0.520
	*fimACDH*	194 (98.0)	117 (100.0)	311 (98.7)	0.154
	*focAC/D*	70 (35.4)	34 (29.1)	104 (33.0)	0.251
	*sfaSAF*	22 (11.1)	17 (15.5)	39 (12.4)	0.373
Non-Fimbrial	*csgABC/DEFG*	198 (100.0)	117 (100.0)	315 (100.0)	-
	*fdeC*	155 (78.3)	96 (82.1)	251 (79.7)	0.422
	*iha*	10 (5.1)	13 (11.1)	23 (7.3)	**0.046**
	*afaE-I*	2 (1.0)	-	2 (0.6)	0.394
	*draAD*	3 (1.5)	-	3 (1.0)	0.247
	*flgBCDEFGHIJ*	198 (100.0)	117 (100.0)	315 (100.0)	-
Siderophore systems	*ent/fep/fes*	197 (99.5)	117 (100)	314 (99.7)	0.629
	*irp1 + 2/fyuA*	168/167 (84.8/84.3)	101/102 (86.3/87.2)	269 (85.4)	0.491
	*iroBCDEN*	144 (72.7)	98 (83.8)	242 (76.8)	**0.025**
	*iuc/iutA*	28 (14.1)	20 (17.1)	48 (15.2)	0.481
	*chuA/hma*	160/110 (80.8/55.6)	97/84 (82.9/71.8)	257/194 (81.6/61.6)	0.643/**0.004**
	*sitABCD*	114 (54.0)	62 (53.0)	176 (55.9)	0.429
Toxins	*cnf1*	107 (54.0)	75 (64.1)	182 (57.8)	0.081
	*vat*	144 (72.7)	87 (74.4)	231 (73.3)	0.752
	*pic*	41 (20.7)	60 (51.3)	101 (32.1)	<**0.001**
	*astA*	25 (12.6)	9 (7.7)	31 (9.8)	0.173
	*cdtB*	13 (6.6)	15 (12.8)	28 (8.9)	0.059
	*hlyCABD*	97 (49.0)	49 (41.9)	146 (46.3)	0.221
	*tosA*	102 (51.5)	63 (53.8)	165 (52.4)	0.689
Autotransporter proteins	*agn43*	77 (38.9	37 (31.6)	114 (36.2)	0.195
	*upaC*	69 (34.8)	69 (59.0)	138 (43.8)	<**0.001**
	*sat*	9 (4.5)	8 (6.8)	17 (5.4)	0.384
Surface polysaccharides	*kpsMTII*	80 (40.4)	69 (59.0)	149 (47.3)	<**0.001**
	*neuC*	15 (7.6)	7 (6.0)	22 (7.0)	0.592
	*neuS*	1 (0.5)	1 (0.9)	2 (0.6)	0.606
Invasins	*ompA*	198 (100.0)	117 (100.0)	315 (100.0)	-
	*ibeA*	84 (42.4)	30 (25.6)	114 (36.2)	**0.003**
	*traT*	69 (34.8)	41 (35.0)	110 (34.9)	0.972
Miscellaneous or unknown function	*malX*	198 (100.0)	117 (100.0)	315 (100.0)	-
	*ompT*	174 (87.9)	107 (91.5)	281 (89.2)	0.323
	*usp*	149 (75.3)	90 (76.9)	239 (75.9)	0.738

^1^ The occurrence of VAGs between animal species was assessed using Chi-square tests, and a value of *p* < 0.05 was seen as statistically significant (bold); *papGII/papGIII*, P fimbriae; *fimACDH*, type 1 fimbriae; *focAC/D*, F1C fimbriae; *sfaSAF*, S fimbriae (sialic acid-specific); *csgABC/DEFG*, curli; *fdeC*, factor adherence *E. coli*; *iha*, iron-regulated-gene-homologue adhesin; *afaE-I*, afimbrial adhesin; *draAD*, dr antigen-binding adhesin; *ent/fep/fes*, entero-bactin synthesis/receptor; *irp/fyuA*, yersiniabactin synthesis/receptor; *iroBCDE/N*, salmochelin synthesis/receptor; *iuc/iutA*, aerobactin synthesis/receptor; *chuA/hma*, heme receptor; *sitABCD*, salmonella iron transporter; *cnf1*, cytotoxic necrotizing factor 1; *vat*, vacuolating autotransporter toxin; *pic*, serin protease autotransporter; *astA*, enteroaggregative *E. coli* toxin; *cdtB*, cytolethal distending toxin; *hlyCABD*, hemolysin A; *tosA*, putative repeat-in-toxin protein; *agn43*, antigen 43; *upaC*, uropathogenic *E. coli* autotransporter; *sat*, secreted autotransporter toxin; *kpsMTII*, group II capsule synthesis; *neuC/S*, K1 capsular polysaccharide; *ompA*, outer membrane protein A; *ibeA*, invasion of brain endothelium; *traT*, serum-resistance associated; *malX*, pathogenicity island marker; *ompT*, outer membrane protein T; *usp*, uropathogenic specific protein.

**Table 3:** Distribution of AMR genes in 315 *E. coli* isolates from urine samples of 117 cats and 198 dogs, differentiated by phylogroup B2 (n = 245) and non-B2 (n = 70) isolates.

Antibiotic	Gene	Cat n (%)	Dog n (%)	*p-*Value ^1^	B2 n (%)	non-B2 n (%)	*p-*Value ^2^	All n (%)
Amino- glycoside	*aac(3)-IId*	-	4 (2.0)	0.301	3 (1.2)	1 (1.4)	1	4 (1.3)
	*aadA*	6 (5.1)	22 (11.1)	0.100	13 (5.3)	15 (21.4)	<**0.0001**	28 (8.9)
	*ant(2* *″* *)-Ia*	1 (0.9)	-	0.371	-	1 (1.4)	0.222	1 (0.3)
	*aphAI-IAB*	32 (27.4)	52 (26.3)	0.895	50 (20.4)	34 (48.6)	<**0.0001**	84 (26.7)
Quinolone	*aac(6* *′* *)-Ib-cr*	1 (0.9)	-	0.371	-	1 (1.4)	0.222	1 (0.3)
	*qnrB*	-	2 (1.0)	0.532	-	2 (2.9)	**0.049**	2 (0.6)
	*qnrS*	1 (0.9)	2 (1.0)	1	-	3 (4.3)	0.089	3 (1.0)
Rifampicin	*arr-3*	1 (0.9)	-	0.371	-	1 (1.4)	0.222	1 (0.3)
β-Lactams	*bla*_TEM_	23 (19.7)	36 (18.2)	0.766	36 (14.7)	23 (32.9)	**0.002**	59 (18.8)
	*bla*_OXA-1_	2 (1.7)	3 (1.5)	1	1 (0.4)	4 (5.7)	**0.009**	5 (1.6)
	*bla*_CTX-M-15_	-	3 (1.5)	0.297	-	3 (4.3)	**0.011**	3 (1.0)
	*bla*_CTX-M-27_	1 (0.9)	-	0.371	-	1 (1.4)	0.222	1 (0.3)
	*bla*_DHA-1_	-	1 (0.5)	1	-	1 (1.4)	0.222	1 (0.3)
	*bla*_CMY-2_	1 (0.9)	-	0.371	1 (0.4)	-	1	1 (0.3)
	*bla*_OXA-48_	1 (0.9)	-	0.371	1 (0.4)	-	1	1 (0.3)
	*bla*_SHV-1_	3 (2.6)	2 (1.0)	0.364	5 (2.0)	-	0.590	5 (1.6)
Phenicols	*catA1*	-	6 (3.0)	0.088	4 (1.6)	2 (2.9)	0.618	6 (1.9)
	*catB3*	1 (0.9)	-	0.371	-	1 (1.4)	0.222	1 (0.3)
	*cmlA1*	-	2 (1.0)	0.532	1 (0.4)	1 (1.4)	0.396	2 (0.6)
	*floR*	3 (2.6)	3 (1.5)	0.674	-	6 (8.6)	<**0.0001**	6 (1.9)
Folate pathway inhibitors	*sul1*	6 (5.1)	14 (7.1)	0.495	10 (4.1)	10 (14.3)	**0.002**	20 (6.3)
	*sul2*	15 (12.8)	22 (11.1)	0.649	18 (7.3)	19 (27.1)	<**0.0001**	37 (11.7)
	*sul3*	-	4 (2.0)	0.301	1 (0.4)	3 (4.3)	**0.036**	4 (1.3)
	*drfA*	12 (10.3)	28 (14.1)	0.383	14 (5.7)	26 (37.1)	<**0.0001**	40 (12.7)
Tetracycline	*tet(A)*	8 (6.8)	21 (10.6)	0.264	17 (6.9)	12 (17.1)	**0.009**	29 (9.2)
	*tet(B)*	5 (4.3)	7 (3.5)	0.741	4 (1.6)	8 (11.4)	<**0.0001**	12 (3.8)
	*tet(D)*	1 (0.9)	-	0.371	1 (0.4)	-	1	1 (0.3)
	*tet(M)*	-	1 (0.5)	1	1 (0.4)	-	1	1 (0.3)
Macrolides	*mph(A)*	1 (0.9)	6 (3.0)	0.265	4 (1.6)	3 (4.3)	0.186	7 (2.2)
	*mph(B)*	-	1 (0.5)	1	1 (0.4)	-	1	1 (0.3)
	*mph(E)*	-	1 (0.5)	1	-	1 (1.4)	0.222	1 (0.3)
	*msr(E)*	-	1 (0.5)	1	-	1 (1.4)	0.222	1 (0.3)
Colistin	mcr-4.6 *	-	1 (0.5)	1	1 (0.4)	-	1	1 (0.3)
Fosfomycin	*fosA7*	3 (2.6)	-	0.050	-	3 (4.3)	**0.011**	3 (1.0)
AMR genes total		128	245	0.244	287	186	<**0.0001**	373

The occurrence of AMR genes was compared among animal species ^1^ and among phylogroups (B2 versus non-B2) ^2^ by employing Chi-square tests. Significance was set at *p* = 0.05 (bold). * Mutations in the *pmrA* and *pmrB* genes that are known to confer colistin resistance were not detected.

**Table 4:** Origin, haemolytic phenotype, molecular characteristics, AMR profiles and AMR genes of CP/ESBL/AmpC-positive *E. coli* isolates.

Sample ID	Animal	Haemolysis	ST	Phylo-Group	CP/ESBL/ AmpC-Positive Isolate	AMR Genes	Mutations			
							*ampC* Promotor	*gyrA*	*parC*	*parE*
IHIT41651	dog	0	410	C	ESBL	*bla*_CTX-M-15_, *bla*_TEM-1B,_*aadA2*, *sul1*, *dfrA12*, *tet(A)*, *mph(A)*	*-*	S83L, D87N	S80I	S458A
IHIT41754	dog	0	1844	B1	AmpC	*bla*_DHA-1_, *bla*_TEM-1B,_*sul1*, *dfrA17*, *tet(B)*, *qnrB4*, *mph(A)*	−1C > T, −18G > A	-	-	-
IHIT42192	cat	0	533	B1	ESBL	*bla*_CTX-M-27,_*sul2*, *dfrA36*, tet(B), sul2	−1C > T −18G > A	D87N, S83L	S80I	
IHIT42968	dog	0	58	B1	AmpC	*bla*_EC11_-like, *dfrA5*	−42C > T * −18G > A −1C > T	-	-	-
IHIT43081	cat	1	372	B2	CP	*bla*_OXA-48_	*-*	-	-	-
IHIT43540	dog	0	88	C	ESBL	*bla*_CTX-M-15,_*aadA1*, *sul2, dfrA1*, *tet(B)*, *qnrS1*	−18G > A −1C > T	S83L	-	-
IHIT43661	cat	1	12	B2	AmpC	*bla*_CMY-2_	*-*	-	-	-
IHIT43802	dog	0	361	A	ESBL	*bla*_CTX-M-15_, *bla*_TEM-32,_*aph(3″)-Ib*, *aph(3′)-Ia, aph(6)-Id*, *sul2, dfrA1*	*-*	D87N, S83L	S80I	S458A
IHIT44091	cat	1	131	B2	AmpC	*bla*_TEM-1B,_*bla*_EC-6_, *aph(3″)-Ib*, *aph(6)-Id*, *sul2*, *dfrA8*	−28G > A	-	-	I529L

AMC: amoxicillin/clavulanic acid; AMP: ampicillin; CFX: cephalexin; CFV: cefovecin; ENR: enrofloxacin; PRA: pradofloxacin; SXT: trimethoprim/sulfamethoxazole; TET: tetracycline; CP: carbapenemase; * associated with phenotypic resistance (ampicillin, ampicillin/clavulanic acid, amoxicillin, amoxicillin/clavulanic acid, cefixime, cefotaxime, cefoxitin, ceftazidime, piperacillin).

## Data Availability

The raw sequencing data are available under NCBI BioProject PRJNA1031431 (https://www.ncbi.nlm.nih.gov/bioproject/1031431, accessed on 24 October 2023).

## Supplementary Materials

The following supporting information can be downloaded at: https://www.mdpi.com/article/10.3390/antibiotics13010038/s1, Table S1: Newly assigned STs, Table S2: Virulence associated genes, Table S3: Genotypic and phenotypic AMR.
