# Supplementary material for: Genotypic Characterization of Uropathogenic Escherichia coli from Companion Animals: Predominance of ST372 in Dogs and Human-Related ST73 in Cats
Source: Antibiotics (Basel). 2023 Dec 30;13(1):38. doi: 10.3390/antibiotics13010038 (PMC10812829; doi:10.3390/antibiotics13010038)
Supplement: Supplementary file 1 [file antibiotics-13-00038-s001.zip › Table S1 Newly assigned STs.pdf]

Name	Host species	*adk*	*fumC*	*gyrB*	*icd*	*mdh*	*purA*	*recA*	New ST	SLV
IHIT43275	cat	1214*	35	2	27	5	16	182	**ST14533**	-
IHIT42530	cat	13	38	19	13	17	28	66	**ST15094**	-
IHIT41828	cat	36	2397*	9	13	17	11	25	**ST15095**	ST73
IHIT42925	dog	13	52	10	14	17	25	1190*	**ST15097**	ST141
IHIT43803	cat	36	24	9	13	1481*	11	25	**ST15098**	ST73
IHIT43020	cat	36	2399*	9	13	17	11	25	**ST15099**	ST73
IHIT43987	dog	10	11	4	1227*	8	13	73	**ST15100**	ST10
IHIT41749	dog	88	103	1514*	36	23	44	26	**ST15102**	ST372
IHIT43030	cat	13	2401*	19	13	30	25	29	**ST15104**	ST372
IHIT41975	dog	88	103	19	1904*	23	44	26	**ST15106**	ST83

Supplemental Table S1. New sequence types (ST) isolated from canine and feline urine in this study and added to EnteroBase. New alleles are indicated with an asterisk (*). If strains are a single locus variant (SLV) of a known ST, this is displayed in the last column.
